# Age-Dependent Impairment of Neurovascular and Neurometabolic Coupling in the Hippocampus

**DOI:** 10.3389/fphys.2018.00913

**Published:** 2018-07-17

**Authors:** Cátia F. Lourenço, Ana Ledo, Miguel Caetano, Rui M. Barbosa, João Laranjinha

**Affiliations:** ^1^Center for Neuroscience and Cell Biology, University of Coimbra, Coimbra, Portugal; ^2^Faculty of Pharmacy, University of Coimbra, Coimbra, Portugal

**Keywords:** aging, nitric oxide, neurovascular coupling, neurometabolism, oxidative stress, hippocampus

## Abstract

Neurovascular and neurometabolic coupling are critical and complex processes underlying brain function. Perturbations in the regulation of these processes are, likely, early dysfunctional alterations in pathological brain aging and age-related neurodegeneration. Evidences support the role of nitric oxide (•NO) as a key messenger both in neurovascular coupling, by signaling from neurons to blood vessels, and in neurometabolic coupling, by modulating O_2_ utilization by mitochondria. In the present study, we investigated the functionality of neurovascular and neurometabolic coupling in connection to •NO signaling and in association to cognitive performance during aging. For this, we performed *in vivo* simultaneous measurements of •NO, O_2_ and cerebral blood flow (CBF) in the hippocampus of F344 rats along chronological age in response to glutamatergic activation and in correlation with cognitive performance. Firstly, it is evidenced the temporal sequence of events upon glutamate stimulation of hippocampal *dentate gyrus*, encompassing the local and transitory increase of •NO followed by transitory local changes of CBF and *p*O_2_. Specifically, the transient increase of •NO is followed by an increase of CBF and biphasic changes of the local *p*O_2_. We observed that, although the glutamate-induced •NO dynamics were not significantly affected by aging, the correspondent hemodynamic was progressively diminished accompanying a decline in learning and memory. Noteworthy, in spite of a compromised blood supply, in aged rats we observed an increased *Δp*O_2_ associated to the hemodynamic response, suggestive of a decrease in the global metabolic rate of O_2_. Furthermore, the impairment in the neurovascular coupling observed along aging in F344 rats was mimicked in young rats by promoting an unbalance in redox status toward oxidation via intracellular generation of superoxide radical. This observation strengthens the idea that oxidative stress may have a critical role in the neurovascular uncoupling underlying brain aging and dysfunction. Overall, data supports an impairment of neurovascular response in connection with cognition decline due to oxidative environment-dependent compromised •NO signaling from neurons to vessels during aging.

## Introduction

The brain is an organ with high metabolic and energetic requirements, and despite representing only 2% of the total body weight, the adult brain receives over 15% of the cardiac output and consumes more than 20% of the body’s total glucose and oxygen sources (Rolfe and Brown, 1997). Further considering the limited reserves and the high energetic demands, it sounds clear that brain function is critically dependent on adequate blood supply and proper delivery of bioenergetic substrates to support its information processing capabilities through neurotransmission (Zlokovic, 2011). As such, the local intensity of neuronal activity continuously determines both the dynamic regulation of cerebral blood flow (CBF) – neurovascular coupling – and utilization of glucose and O_2_ by different cell types – neurometabolic coupling (Ledo et al., 2017). The functional deterioration of these mechanisms is recognized as a pathophysiological feature in brain aging and several age-related neuropathological conditions, such as Alzheimer’s and Parkinson’s disease (Girouard and Iadecola, 2006; Lourenço et al., 2015; Iadecola, 2017). In addition to the observed inverse correlation between resting CBF and chronological age (Fisher et al., 2013; Desjardins et al., 2014; Tarumi and Zhang, 2017), several studies support the occurrence of reduced hemodynamic responses coupled to neuronal activation during non-pathological aging, both in animals and humans (Park et al., 2007; Gauthier et al., 2013; Fabiani et al., 2014; Toth et al., 2014; Balbi et al., 2015; Jessen et al., 2015; Lourenço et al., 2017). Although a matter of debate, it has also been suggested that cerebral energy metabolism (including both the transport and utilization of oxygen and glucose) is altered during aging (Yin et al., 2016).

Nitric oxide (•NO) is a free radical signaling molecule produced by a family of enzymes – nitric oxide synthase (NOS) - that catalyze the conversion of L-arginine to L-citrulline and •NO using O_2_ and NADPH (Alderton et al., 2001). In the brain, it can be produced within the neurovascular unit either by neuronal, glial or vascular cells, being implicated in several physiological mechanisms, such as synaptic plasticity, modulation of neurotransmitter release, and regulation of CBF (Dawson and Dawson, 1998). In particular, the •NO produced by the neuronal isoform of NOS (nNOS) upon stimulation of glutamatergic neurotransmission plays a pivotal role in neurovascular coupling in the hippocampus, acting as a direct signaling messenger from active neurons to blood vessels, thus promoting vasodilation (Lourenço et al., 2014b). A key tenet of •NO bioactivity is that besides participating in important physiological functions, it can has also be involved in several pathological mechanisms that actively contribute to neurodegeneration in aging and age-related neuropathological conditions (Yuste et al., 2015). This deviation in •NO bioactivity may occur as a consequence of a shift in the redox environment toward more oxidizing conditions. These conditions foster the generation of reactive oxygen and nitrogen species (RONS) capable of interacting with a wide range of molecules, modifying several signaling pathways and ultimately leading to cell damage (Knott and Bossy-Wetzel, 2009).

While several studies have demonstrated age-dependent alterations in •NO signaling, CBF, brain metabolism and cognitive decline, the putative connection and the temporal pattern between these factors still requires demonstration. In this work, we aimed to investigate whether brain aging is accompanied by impairment in neurovascular and neurometabolic coupling in the rodent hippocampus, exploring the correlation with learning and memory performance and the glutamate-•NO signaling pathway. Furthermore, we explored the putative role of increased oxidative stress in contributing to the neurovascular uncoupling. To this purpose, we performed a cross-sectional study in a rodent model of aging, encompassing behavior testing of cognitive performance and *in vivo* simultaneous measurements of •NO, O_2_ and CBF upon glutamatergic activation in the hippocampus (*dentate gyrus*) of young, middle- and old-aged Fischer 344 rats.

## Materials and Methods

### Chemicals and Solutions

All chemicals used were analytical grade and obtained from SigmaAldrich, unless otherwise stated. Phosphate buffer (0.05 M PBS) was prepared in MilliQ water and had the following composition (in mM): 10 NaH2PO4, 40 Na2HPO4, and 100 NaCl (pH 7.4). A saturated •NO solution was prepared in a Vacutainer^®^ containing MilliQ water purged with Nitrogen (AirLiquid) and then bubbled for 30 min with •NO (AirLiquid) gas pre-cleaned by passage in NaOH pellets and 5M NaOH solution, essentially as previously described (Barbosa et al., 2008). The final concentration of this solution has been determined to be 1.8 mM (Zacharia and Deen, 2005). A saturated O_2_ solution was prepared in a vacutainer containing PBS Lite bubbled for 30 min with Cabox (95%O_2_/5%CO_2_ gas mixture, Linde) as described previously (Lourenço et al., 2017). In accordance to Henry’s Law, the final concentration of O_2_ at room temperature is 1.3 mM (Sander, 1999).

### Animals

All the procedures used in this study were performed in accordance with the European Union Council Directive for the Care and Use of Laboratory animals, 2010/63/EU and approved by local ethics committee (ORBEA). Experiments were conducted in male Fischer 344 rats acquired from Charles River Laboratories (Barcelona, Spain) at 2 months of age and maintained in the animal house facilities of the Center for Neuroscience and Cell Biology (Coimbra). Animals were randomly separated into 3 groups: young (4–6 months old), middle aged (12 months old) and old aged (18 to 23 months old). They were housed in pairs in filter-topped type III Makrolon cages allocated in a room with controlled environment: a temperature of 22–24°C, relative humidity of 45–65%, 15 air exchanges per hour and a 12:12 light/dark cycle. Animals were fed a standard chow rat diet (4RF21-GLP Mucedola, SRL, Settimo Milanese, Italy) and were provided chlorinated water *ad libitum*. Cage bedding (standard corn cob) was changed three times a week and environmental enrichment was provided with tissue paper and a cardboard tube. The experiments dedicated to the impact of oxidative stress in the neurovascular coupling were conducted in young Wistar rats (3–4 months old) maintained in the same conditions.

### Behavior Testing of Memory Performance

The spatial reference memory version of the Morris water maze was performed in a large circular tank (1.5 m diameter, 50 cm depth) filled with water maintained at 22°C. A transparent Plexiglass platform (10 cm diameter) was submerged 1 cm beneath the water surface and maintained in a constant position (centered in an imaginary quadrant). Distinctive visual cues were set up on the wall surrounding the tank, positioned in the midpoint of the perimeter of each quadrant. The training session consisted of 5 consecutive trials during which rats, following a pseudo-random order, were placed in the water facing the tank at the points defined by the quadrants limits. Animals were allowed 60 s to locate the hidden platform, and in case of failure, were guided by the investigator to the platform. In either case, animals were left on the platform for 10 s before being removed from the tank. The latency to find the platform was recorded for each trial. The retention of the spatial training was then assessed after 24 h in a single probe trial consisting of a 60 s free swim in the tank without the platform. The number of crossings over the platform location and the percentage of time spent in the quadrant opposite to the target quadrant was determined as an index of memory performance.

### *In Vivo* Setup for Simultaneous Measurement of •NO, O_2_ and Cerebral Blood Flow

**FIGURE 1:**
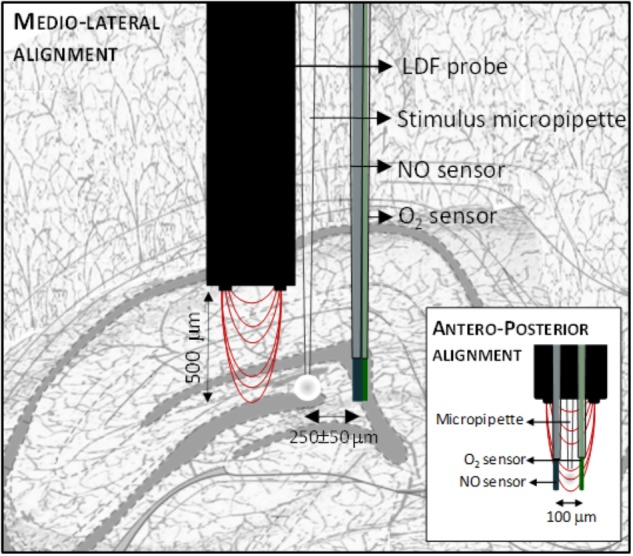
Schematic representation of the array used to simultaneously measure in •NO, Cerebral Blood Flow and O_2_ in the *dentate gyrus* of the hippocampus. The array comprises a microsensor for •NO, a microsensor for O_2_, a microinjection pipette and a laser Doppler flow probe assembled in the represented geometry.

The •NO, O_2_ and CBF dynamics were measured essentially as previously described (Lourenço et al., 2014b). Both •NO and O_2_ measurements were performed using modified carbon fiber microelectrodes (30 μm Ø fiber, Textron Lowell, MA, United States). •NO microelectrodes were modified with Nafion^®^ and *o*-phenylenediamine to improve their analytical properties for •NO *in vivo* measurements. Each sensor was evaluated for sensitivity and selectivity against major interferents (ascorbate, nitrite and dopamine) by amperometry at +0.9 V vs. Ag/AgCl/KCl 3M. For O_2_ measurements, carbon fiber microelectrodes were coated with a composite film of multi-wall carbon nanotubes (10 mg/ml) in 0.5% Nafion^®^ and their sensitivity was evaluated by amperometry at -0.55 V vs. Ag/AgCl/KCl 3M. All electrochemical recording were performed using a FAST16mkII bipotentiostat (Quanteon, LLC, Nicholasville, KY, United States) in a two-electrode configuration, as previously described (Barbosa et al., 2008). CBF was measured using a laser Doppler flowmeter device (Periflux system 5000, Perimed, Sweden) coupled with a needle probe (PF411; outer diameter, 450 μm; fiber separation, 150 μm; wavelength, 780 nm). The •NO and O_2_ microsensors and the Laser Doppler probe were assembled to an ejection micropipette using sticky wax in a predefined geometric configuration as represented in **Figure 1**. The micropipette was filled with 20 mM L-glutamate prepared in NaCl 0.9% using a syringe fitted with a flexible microfilament (MicroFil, World Precision Instruments, United Kingdom) prior to insertion into the brain.

Rats were anesthetized with isoflurane (induction at 5% and maintenance at 1.5 – 2%) carried in medicinal oxygen (Conoxia, Linde) using an E-Z Anesthesia vaporizer (Braintree Scientific, Inc., United States). Following induction, the animal was placed in a stereotaxic apparatus and body temperature was maintained at 37°C with a heated pad coupled to a Gaymar Heating Pump (Braintree Scientific, Inc., United States). A midline incision was made with a scalp, the skin was reflected and a hole was drilled through the skull, exposing the brain surface overlying the hippocampus. Another hole was drilled in a site remote from the recording area for insertion of a pseudo-reference electrode obtained by produced by electro-oxidation of the exposed tip of a Teflon-coated Ag wire (200 μm o.d., Science Products GmbH, Hofheim, Germany) in 1M HCl saturated with NaCl. After removing the dura matter, the array was inserted into the hippocampus according to coordinates calculated based on the rat brain atlas (Paxinos and Watson, 2007). After the insertion of the array into the hippocampus, it was allowed to stabilize for 20 min. Glutamate was locally delivered by pressure ejection (1 s, 7–15 psi) using a Picospritzer III (Parker Hannifin Corp., General Valve Operation, United States).

To address the impact of oxidative stress in the neurovascular coupling in young Wistar rats, DMNQ (2,3-dimethoxy-1,4-naphthoquinone) was administered by intracerebroventricular injection (200 nmol in 5 μL of saline) between consecutive glutamate stimulations and the effects evaluated after 30 min, as previously described (Lourenço et al., 2014b).

### Protein Blotting Analysis

Dissected hippocampi were homogenized in a potter S homogenizer (2 mL capacity) in 0.4 mL of buffer containing NaCl 150 mM, NP40 1% (v/v), sodium deoxycholate (DOC) 0.5% (w/v), sodium dodecyl sulphate (SDS) 0.1% (w/v), Tris-HCl 50 mM (pH 7.4) and protease inhibitor cocktail 0.1% (v/v). Homogenates were centrifuged at 14000 rpm for 15 min at 4°C and the protein content of the supernatants was quantified using the Bradford Bio-Rad Protein Assay (Bio-Rad, Portugal). Protein extracts (40 μg of protein) were fractionated onto 10% SDS-polyacrylamide gels and transferred to polyvinylidene difluoride (PVDF) membranes (GE Healthcare, United Kingdom). Western blots were probed with rabbit anti-nNOS polyclonal antibody (Chemicon, Millipore, United States) overnight at 4°C. The bound antibody was detected by alkaline phosphatase-conjugated secondary anti-rabbit antibody (Abcam, United Kingdom) (1/20000 in TBS-T with 1% BSA, 1 h, room temperature), revealed using an enhanced chemifluorescence (ECF) kit (GE Healthcare, United Kingdom) and visualized in a VersaDoc 3000 (Bio-Rad, Portugal). β-Actin was used as control for protein loading.

### Data Analysis

The •NO/O_2_ and CBF recordings were synchronized using OriginPro 7.5 based on markers extracted from the respective recordings. The •NO were characterized in terms of peak amplitude of the signal, based on the conversion of the amperometric currents to fluxes according to Faraday’s law (*I*= n.F.Φ, in which *I* corresponds to the amperometric current, *n* corresponds to the one electron per molecule exchanged for the oxidation of •NO, *F* corresponds to the Faraday constant and Φ is the flux). The O_2_ dynamics were characterized in terms of maximal amplitude of decrease and increase phases of the glutamate-induced response, based on the conversion of the amperometric currents to *p*O_2_ according to microsensors sensitivity. The CBF changes were characterized in terms of amplitude change (relative to pre-stimulation CBF basal levels). To perform the cross-correlation analysis, the time series of O_2_ and CBF recordings were matched and the phase difference between the time courses was characterized by the time corresponding to the maximum of the cross-correlation coefficient in each period. A linear correlation analysis was used to estimate the correlation between the increase in •NO and CBF or between CBF and O_2_. For protein blotting analysis, the relative intensities of protein bands were analyzed using the Image J Software. All data are presented as mean ± SEM. Statistical analysis of the data was performed using the GraphPad Software applying ordinary one-way analysis of variance (ANOVA) followed by *post hoc* Bonferroni Multiple Comparison Test or Kruskal–Wallis test followed by Dunn’s multiple comparisons test. The statistical significance was considered at *p* < 0.05.

## Results

### F344 Rats Show Age-Dependent Decline in Spatial Memory

**FIGURE 2:**
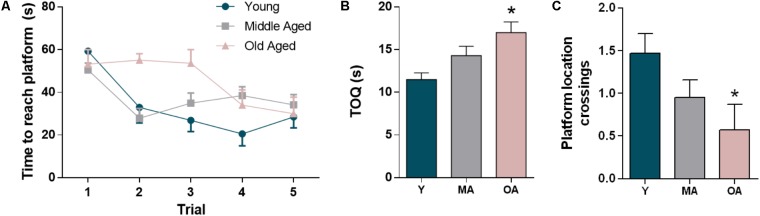
Evaluation of age-dependent decline in short-term memory in the F344 rats. The memory impairment is evidenced by the escape latencies in the training trials **(A)** and the percentage of time spent in the quadrant opposite to the target quadrant **(B)** and the crossings in the platform location **(C)** in the 24-h retention probe test. All the values represent the mean ± SEM. ^∗^*p* < 0.05.

The Morris water maze test was used to assess decline in spatial reference memory, a form of working memory associated with hippocampal function (Vorhees and Williams, 2006). As expected, F344 rats showed decline of both learning and memory capability along age progression, as evidenced by the score of the parameters evaluated both in the acquisition test and in the 24-h retention probe trial (**Figure 2**). In the acquisition test, young and middle-aged animals showed the expected learning curve, with progressive decrease in escape latency is successive trials, while comparatively the old aged animals displayed a delay in learning the localization of the hidden platform (**Figure 2A**). The ability to remember the previous platform location 24-h later was also significantly affected by aging, as reflected by the increase in time spent in the quadrant opposite to the target quadrant and the lower crossings over the platform location. Middle and old aged rats spent an increasing time in the opposite quadrant as compared to young animals (24 and 47%, respectively) (**Figure 2B**). Also, we observed a decrease in the number of crossings over the platform location for the middle and old ages groups when compared to the young group (35 and 61% respectively, **Figure 2C**). These results point toward a progressive age-dependent impairment of spatial reference memory.

### The Coupling Between Nitric Oxide Dynamics, Cerebral Blood Flow and *p*O_2_ Is Impaired With Aging in F344 Rats

**FIGURE 3:**
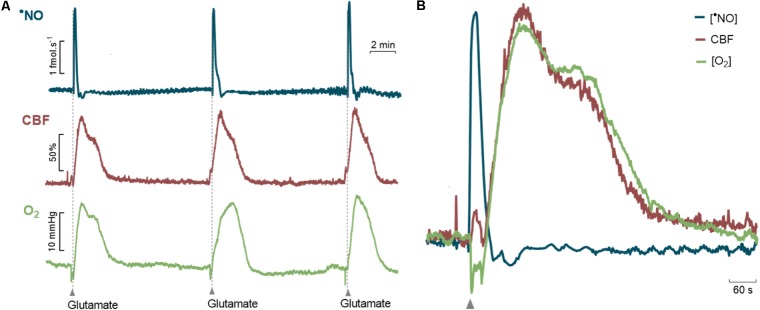
Glutamate-induced changes in •NO, Cerebral Blood Flow and O_2_ in the *dentate gyrus* of the hippocampus of anesthetized F344 rats. **(A)** Representative recording of amperometric signals of •NO (blue line), cerebral blood flow (red line) and amperometric signals of O_2_ (green line) during glutamatergic activation. Glutamate (0.5 nmol, 1 s) was locally applied by pressure ejection at the time indicated by the upward arrows. **(B)** Detailed temporal correlation between the dynamic changes in the •NO concentration, CBF and O_2_ tension.

Measurements of CBF, •NO and O_2_ were made simultaneously in the *dentate gyrus* of the hippocampus of anesthetized F344 rats at different ages in response to glutamate stimulations. In all groups, the local injection of glutamate induced transitory changes in •NO, CBF, and O_2_ with predictable temporal correlation between the three events (**Figure 3**). Specifically, the glutamate-induced increase in •NO was followed by a transient increase in CBF, consistent with the role of neuronal-derived •NO as a mediator of neurovascular coupling in this brain region (Lourenço et al., 2014b). It is of note that, following •NO increase, biphasic changes in the local *p*O_2_ were characterized by an initial decrease matching the time course of •NO dynamics and a later increase matching the time course of CBF increase (**Figure 3B**).

**FIGURE 4:**
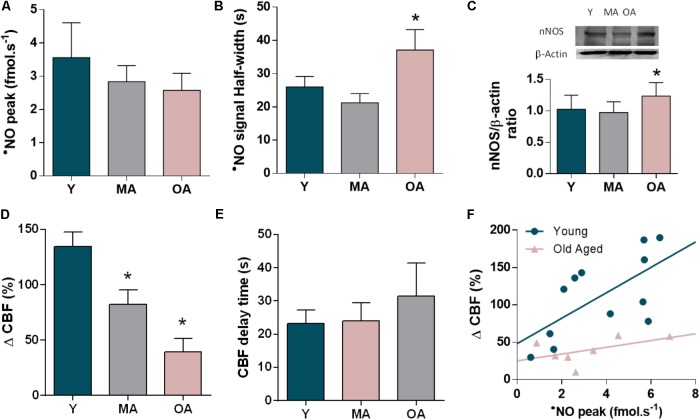
Changes in the •NO-mediated neurovascular coupling along age progression in the *dentate gyrus* of hippocampus of F344 rats. **(A)** Peak amplitude and **(B)** signal duration of the •NO concentration dynamics elicited by glutamate in young (Y), middle aged (MA), and old aged (OA) F344 rats. **(C)** Western Blot of nNOS in hippocampus of Y, MA and OA rats and quantitative analysis of nNOS normalized to β-actin levels. **(D)** Amplitude of the CBF changes coupled to •NO concentration dynamics along age and **(E)** delay period for CBF increase following •NO rise. Each bar represents mean ± SEM. ^∗^*p* < 0.05. **(F)** Correlation between the amplitudes of •NO and CBF changes in young (blue) and old aged (pink) F344 rats (*R*^2^ = 0.428, *p* = 0.021 in young animals; *R*^2^ = 0.267, *p* = 0.234 in old-aged animals).

Quantitatively, the peak [•NO] resulting from glutamate stimulation showed a tendency to decline with age (28% old vs. young, *p*= 0.432) (**Figure 4A**). In turn, the •NO signal showed slower kinetics in older animals as compared to young animals, reflected in a significant increased signal half-width (43% old vs. young, *p*= 0.015) (**Figure 4B**). This quantitative profile of •NO concentration gradients in the *dentate gyrus* occurs along with a slight increase in nNOS expression levels in the whole hippocampus in old aged animals (20% old vs. young, *p* = 0.038) (**Figure 4C**). Conversely, the hemodynamic response coupled to the •NO dynamics decreased progressively with aging, as shown in **Figure 4D** (71% old vs. young, *p*< 0.001). In aged animals, the impaired CBF change is characterized by an increase in the delay period between •NO rise and onset of CBF response (**Figure 4E**). Noteworthy, the linear correlation between •NO production and CBF observed in young animals is lost for the older animals (*R*^2^ = 0.428, *p* = 0.021 in young animals; *R*^2^ = 0.267, *p* = 0.234 in old aged animals) (**Figure 4F**).

**FIGURE 5:**
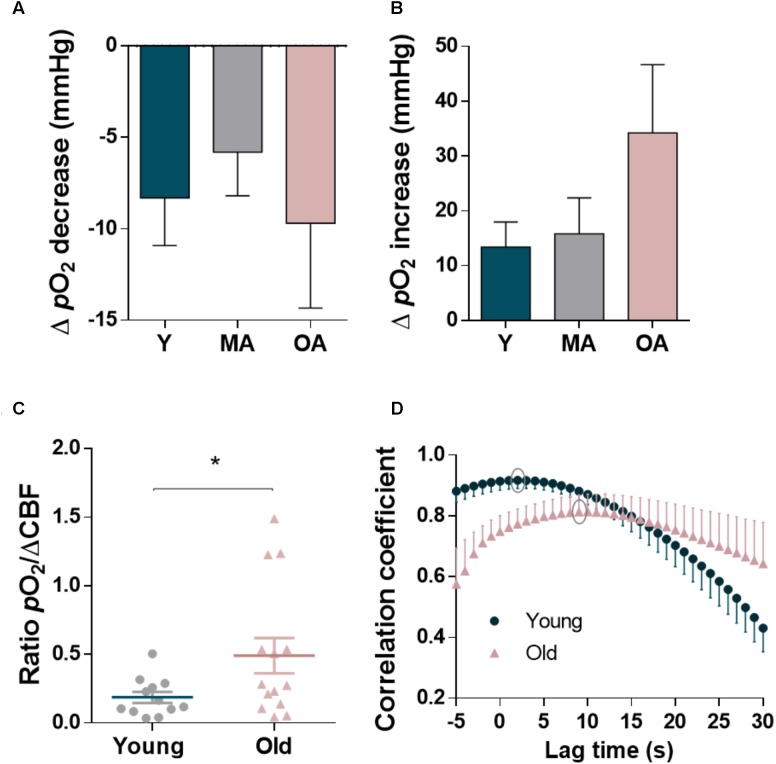
Quantitative analysis of biphasic changes in oxygen tension coupled to the glutamatergic activation in the dentate gyrus of hippocampus of F344 rats along aging and relationship with cerebral blood flow changes. **(A)** Average decrease in Δ*p*O_2_ in the initial component of the signal and **(B)** average increase in Δ*p*O_2_ in the later phase coupled to the CBF increase. Each bar represents mean ± SEM. ^∗^*p* < 0.05. **(C)** Ratio of the *p*O_2_ change to the CBF change in young and old-aged animals. **(D)** Cross-correlation analysis of the time course of CBF and O_2_ dynamics elicited by glutamate in young and old-aged animals. Average strongest correlation (grey circles) was found at 2 s and 9 s delay of O_2_ relative to CBF in young and old rats, respectively. All the values represent the mean ± SEM. ^∗^*p* < 0.05.

Age also influenced the dynamic changes of local *p*O_2_ resulting from glutamatergic stimulation in the hippocampus, in particular the second phase of the signal that appeared temporally correlated with the hemodynamic response (**Figure 5**). While the amplitude of Δ*p*O_2_ during the initial decrease phase, temporally coupled to the transient increase in •NO, remained similar in the three age groups (**Figure 5A**, *p*= 0.574), the second phase of Δ*p*O_2_ increased significantly in old aged animals (**Figure 5B**, *p*= 0.010), despite the decrease in amplitude of the CBF changes observed at this age. As shown in **Figure 5C**, and as expected, this results in a significant increase of the ratio between *Δp*O_2_ and ΔCBF in old-aged F344 rats as compared to younger animals (*p*= 0.038). Apparently, in the brain of old animals, O_2_, although available, is not used as efficiently as in younger animals. In addition to the quantitative changes, the profile of Δ*p*O_2_ was characterized by an increased delay relative to the hyperemic response. The cross-correlation analysis of the time course of Δ*p*O_2_ and CBF dynamics elicited by glutamate evidenced a temporal shift between Δ*p*O_2_ and CBF dynamics in old animals: the average strongest correlation was found at 2 s for young rats (*R*^2^= 0.917) and at 9 s for old-aged rats (*R*^2^= 0.813) (**Figure 5D**).

Overall, these results point toward an imbalance in the regulation of both neurovascular and neurometabolic coupling during normal aging, in close correlation with the compromised cognitive function.

### The Age-Related Uncoupling Between Nitric Oxide Dynamics and Cerebral Blood Flow Is Mimicked by Oxidative Stress Conditions

**FIGURE 6:**
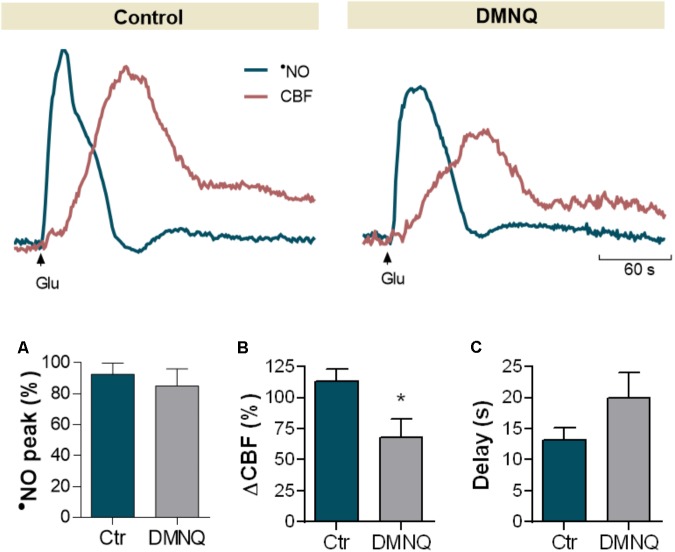
Effect DMNQ, a redox-cycling agent that promotes the intracellular production of superoxide radical, on glutamate-induced •NO signals and CBF changes in the hippocampus of Young Wistar rats. Quantitative analysis of the effect of DMNQ in •NO peak amplitude **(A)**, amplitude in CBF changes **(B)** and delay period for CBF increase following •NO rise **(C)**. Each bar represents mean ± SEM. ^∗^*p* < 0.05.

Oxidative stress has been associated to aging by contributing to the dysfunction of many physiological processes required for proper brain function and to cognitive decline (Perluigi et al., 2014). Hypothesizing that oxidative stress may underlie the observed neurovascular uncoupling during normal aging, we evaluated the effect of increasing the oxidative load on neurovascular coupling in young Wistar rats (**Figure 6**). To test this effect, we used DMNQ, a redox-cycling quinone that triggers intracellular production of superoxide radical (Brunmark and Cadenas, 1989). DMNQ promoted no significant change in the amplitude of glutamate-induced •NO concentration dynamics (**Figure 6A**, *p*= 0.592), but a significant decrease in the coupled CBF changes was observed (**Figure 6B**, 40 ± 14%, *p*= 0.038). Furthermore, we observed a tendency for an increase in the delay period between •NO rise and onset of CBF change following DMNQ as compared to the control signals (**Figure 6C**, *p*= 0.063).

## Discussion

In the present study, we sought to investigate the functionality of neurovascular and neurometabolic coupling in association to cognitive performance during aging in F344 rats. We have performed simultaneous measurements of •NO concentration dynamics, *p*O_2_ and CBF responses associated to glutamatergic activation in the *dentate gyrus* of the hippocampus at three different ages. Our findings revealed age-dependent changes in the dynamic profile of •NO, CBF and *p*O_2_ associated with a decline in learning and memory function evaluated by a spatial reference memory version of the Morris water maze test. While only subtle changes were found in glutamate-elicited •NO concentration dynamics, CBF and *p*O_2_ responses were substantially different between young and old aged rats. Interestingly, we found that although the hemodynamic response coupled to the •NO transients showed an age-dependent decline, this was accompanied by an increase *Δp*O_2_ for this hyperemic phase of the glutamate-evoked response, suggesting a deficient utilization of O_2_ in the aged brain.

We have previously described age-dependent changes in endogenous •NO concentration gradients linked to glutamatergic activation, characterized by differences in both the amplitude and temporal profile of the •NO transients in different brain regions (Ledo et al., 2015). Curiously, in the hippocampus, the trend observed in the *dentate gyrus* does not exactly match that previously reported for *CA1* subregion. In particular, while an age-dependent decline in both amplitude and signal duration was reported for *CA1*, here in the *dentate gyrus* we observed an age-dependent increase in the duration of •NO transients in spite of the lower amplitude for the same stimulation protocol. The concomitant observation that global nNOS expression levels in the hippocampus is higher in older animals strengths the notion of a very tight and specific regulation of •NO signaling amongst different brain regions, as previously suggested (Lourenço et al., 2014a), and supports that it occurs differently in each brain region with age progression. Also, it reinforces the paramount importance of directly measuring the •NO concentration gradients to infer •NO bioactivity. It is known that the •NO synthesis by nNOS can be modulated by several mechanisms, including the availability of cofactors and substrates, interactions between the enzyme with other proteins, subunit dimerization, post-translational modification (Zhou and Zhu, 2009), as well as by oxidative stress conditions (Sun et al., 2008). For instance, in the hippocampus there is evidence for age-related and subregion-specific changes in the activity of arginase, an enzyme that shares the substrate L-arginine with NOS and has a critical role in the regulation of •NO production (Liu et al., 2003; Gupta et al., 2012). Also, several antioxidant defense mechanisms are suggested to be compromised in aged brain in a region-specific manner (Cardozo-Pelaez et al., 1999; Siqueira et al., 2005).

Conversely to the profile of •NO concentration gradients associated to the glutamatergic activation, the •NO-mediated neurovascular coupling deteriorated significantly with age, as revealed by the significantly decreased amplitude and increased delay of the hemodynamic responses in older animals. This observation corroborates several lines of evidence that support the derailment of neurovascular coupling during non-pathological aging in several animal models (Park et al., 2007; Toth et al., 2014; Balbi et al., 2015; Jessen et al., 2015; Lourenço et al., 2017) as well as in humans (Gauthier et al., 2013; Fabiani et al., 2014). Interestingly, in F344 rats the notorious impairment in neurovascular coupling precedes a significant decline in cognitive function, thus providing support for a prominent role of cerebrovascular dysfunction in the deterioration of neuronal function, as previously demonstrated in a mouse model of Alzheimer’s disease (Lourenço et al., 2017).

The age-dependent neurovascular uncoupling here reported also preceded the impairment in the tissue oxygen utilization, as suggested by the observed changes in the glutamate-evoked responses in tissue *p*O_2_ along aging. The temporal profile of tissue *Δp*O_2_, reflects a moment-to-moment balance between local consumption and supply and the biphasic nature of the signals is supported by the prevalence of each one of these processes in a certain phase: an initial negative component results from the local increase in the rate of O_2_ consumption while the later positive component is a result of the increased supply over demand as a result of the local CBF response (Thompson et al., 2003). We observed that the initial component of *Δp*O_2_ associated with increased O_2_ consumption remained unaltered during aging, while the component of the *Δp*O_2_ correlated with the hyperemic response was significantly increased. This occurred in spite of the compromised CBF response in aged rats, thus suggesting that it is unrelated to increased O_2_ delivery but instead with a decrease in the global metabolic rate of O_2_. Indeed, by using high resolution respirometry in intact hippocampal slices we have previously demonstrated that both basal and maximal O_2_ consumption rates decrease in an age-dependent manner in mice (Dias et al., 2016). Cadenas and coworkers have also demonstrated a reduction in oxidative phosphorylation in F344 rats with aging, linking the impairment of mitochondrial bioenergetics with increased nNOS expression and nitration of relevant mitochondrial proteins (Lam et al., 2009). One would expect that an age-dependent impairment in hippocampal oxidative metabolism be reflected in the two components of the tissue *p*O_2_ dynamics. Yet, this hypothesis cannot be disregarded considering the dynamic nature of the O_2_ response and the likely interaction of both components. For instance, in the young animals the smaller delay in the CBF response may reduce the amplitude of the measured initial dip in *p*O_2_. Also, assuming the age-dependent reduction in O_2_ metabolism, and thus a higher tissue basal *p*O_2_, we would expect a differential profile of the O_2_ consumption related to neuronal activation. Of note, mitochondrial respiration can be decreased in consequence of glutamatergic activation as a result of the reversible and competitive inhibition of with cytochrome *c* oxidase by •NO (Cooper and Giulivi, 2007; Ledo et al., 2010), with the potency of this inhibition being dependent of the O_2_ concentration and the enzyme redox state (Mason et al., 2006).

The mechanisms by which aging impairs neurovascular and neurometabolic coupling are likely multifaceted, but the available evidence increasingly supports the primordial role of oxidative stress in age-dependent dysfunction of these and other mechanisms underlying neurodegeneration (Santos et al., 2013; Lourenço et al., 2015; Yin et al., 2016). Previous research supports age-related oxidative damage to several lipids, proteins, and enzymes in F344 rats (Baek et al., 1999; Navarro et al., 2008; Gilmer et al., 2010), in some cases in correlation with changes in mitochondrial bioenergetics (Long et al., 2009) and cerebrovascular dysfunction (Mayhan et al., 2008). By generating oxidative stress with DMNQ in young Wistar rats we were able to mimic the uncoupling between •NO and CBF dynamics observed in aged F344 rats. This redox-cycling quinone triggers the intracellular production of superoxide that rapidly reacts with •NO in a diffusion-controlled reaction, decreasing •NO bioavailability and generating ONOO^-^, a potent oxidant (Koppenol et al., 1992; Radi et al., 2002). Although no relevant decrease in •NO dynamics was detected, a significant inhibition of CBF changes was observed under these conditions, suggesting an interruption of the signaling from neurons to vessels. Both the relative abundance of •NO and O_2_^-^ at the source (neurons) and effector site (vascular cells) may contribute to the observed changes. In blood vessels, in addition to a lower •NO concentration in consequence of diffusion, a higher steady-state concentration of O_2_^-^ in association to DMNQ treatment is expected as compared to neurons, due to a lower expression of MnSOD (Ruetzler et al., 2001). This would result in a lower •NO bioavailability at the vessel, thus leading to a reduction in the measured hemodynamic response. A caveat should be made regarding the fact that DMNQ experiments were performed in Wistar rats. In spite of a similar profile in the •NO-dependent neurovascular coupling observed in young Wistar and F344 rats, we cannot disregard the potential interstrain differences in the mechanisms underlying age-dependent neurovascular uncoupling and, as such, warn against the direct transposition of the results between both strains. Yet, in general terms, this observation strengthens the idea that oxidative stress may have a critical role in the neurovascular uncoupling observed in aging as suggested by others. For instance, age-dependent neurovascular uncoupling has been shown to be reversed by interventions that improve oxidative status, including the inhibition of NADPH oxidase (Park et al., 2007) and/or mitochondria-derived production of reactive oxygen species (Toth et al., 2014). This observation with DMNQ encourages further studies addressing the impact of oxidative stress over neurometabolic coupling in correlation to glutamatergic activation. Redox proteomic analysis has revealed oxidative damage of several proteins in the aged rodent brain, many of which involved in bioenergetic biochemical pathways such as glycolysis, the tricarboxylic acid cycle and ATP production (Perluigi et al., 2010). Yet, the impact of these modifications over oxidative metabolism during aging remains controversial (Baek et al., 1999; Navarro et al., 2008; Long et al., 2009; Gilmer et al., 2010; Stauch et al., 2015).

In sum, our study demonstrates that non-pathological brain aging involves changes in both neurovascular and neurometabolic function in the hippocampus, in close correlation with compromised cognitive function, suggesting a role for oxidative stress. This expands our understanding of brain aging and contributes toward a comprehensive elucidation of the mechanisms underlying neurodegeneration in neuropathological conditions for which aging is a major risk factor.

## Author Contributions

CL contributed to study design, conducted the experiments, analyzed the data, prepared the figures, wrote and revised the manuscript. AL contributed to study design, conducted the experiments, analyzed the data, and revised the manuscript. MC provided assistance to the experiments and data analysis. RB discussed and revised the manuscript. JL conceived and supervised the study and discussed and revised the manuscript. All authors approved the final version of the manuscript.

## Conflict of Interest Statement

The authors declare that the research was conducted in the absence of any commercial or financial relationships that could be construed as a potential conflict of interest. The handling Editor declared a shared affiliation, though no other collaboration with the authors.
